# Supplementary material for: Impact of COVID-19 on In-Patient and Out-Patient services in Bangladesh
Source: PLoS One. 2025 Feb 28;20(2):e0315626. doi: 10.1371/journal.pone.0315626 (PMC11870377; doi:10.1371/journal.pone.0315626)
Supplement: Attachment — Submitted filename: Plos.one revision.docx [file pone.0315626.s001.docx]

Dear Editor,

I hope this message finds you well. I have reviewed the manuscript you sent and have provided my revision comments below:

1. Title: The title is quite long. A more concise version would be: “Impact of COVID-19 on Healthcare Utilization in Bangladesh: Analysis of Routine Data.”
2. Short Title: Please correct the short title to: “Impact of COVID-19 on In-Patient and Out-Patient Care.”
3. Language: The article needs to be revised by a native English writer to improve readability and clarity.

**Abstract:**

4. Abbreviations: Abbreviations should be defined the first time they are used in the manuscript.

**Introduction:**

5. References: Provide the reference for lines 42-45.
6. References: Provide the reference for lines 70-72.
7. Clarity: The introduction needs to be rewritten to avoid repetition and to be more concise.

**Methods:**

8. Could you please provide a detailed paragraph outlining the assessment methods utilized in your research? This should include a description of both the direct and indirect methods employed, such as tests, surveys, interviews, or observational techniques, and how these methods contributed to the reliability and validity of your findings.

**Data Analysis:**

9. Using the Durbin-Watson statistic to check for autocorrelation is correct. However, note that the Durbin-Watson test is typically used for linear regression models. For Poisson regression, you might consider using other methods like the autocorrelation function (ACF) or partial autocorrelation function (PACF). (line 135)
10. Clarify whether you used Poisson regression or linear regression for the final analysis. If Poisson regression was used, remove the mention of applying a linear regression model. (line 137)

**Results:**

11. Please correct the sentence in lines 147-148 to: “The number of OPD visits fell in 2020 (34.1 million) compared to 2019 but showed an upward trend, reaching the targeted predicted value in 2021.”
12. Correct “increases” to “increased” in line 150.
13. Line 220, table 2: The confidence interval for Dhaka in 2020 is listed as (0.83-0.78), which seems incorrect because the lower bound should be less than the upper bound.

**Discussion:**

14. Please compare your major findings to previous studies found in the literature.
15. Write a small paragraph recommending possible measures to manage such pandemics in the future, while citing experiences from previous pandemics
